# Visual scanpath training to emotional faces following severe traumatic brain injury: A single case design

**DOI:** 10.16910/jemr.14.4.6

**Published:** 2021-10-21

**Authors:** Suzane Vassallo, Jacinta Douglas

**Affiliations:** La Trobe University, Melbourne, Australia; University of Technology, Sydney, Australia; Summer Foundation, Melbourne, Australia

**Keywords:** Eye tracking, scanpath, visual attention, gaze, traumatic brain injury, emotion, facial expression, facial expression recognition training

## Abstract

The visual scanpath to emotional facial expressions was recorded in BR, a 35-year-old male with chronic severe traumatic brain injury (TBI), both before and after he underwent intervention. The novel intervention paradigm combined visual scanpath training with verbal feedback and was implemented over a 3-month period using a single case design (AB) with one follow up session. At baseline BR’s scanpath was restricted, characterised by gaze allocation primarily to salient facial features on the right side of the face stimulus. Following intervention his visual scanpath became more lateralised, although he continued to demonstrate an attentional bias to the right side of the face stimulus. This study is the first to demonstrate change in both the pattern and the position of the visual scanpath to emotional faces following intervention in a person with chronic severe TBI. In addition, these findings extend upon our previous work to suggest that modification of the visual scanpath through targeted facial feature training can support improved facial recognition performance in a person with severe TBI.

## Introduction

Traumatic brain injury (TBI) results from an external force to the head leading to transient or permanent alteration in brain function (3; 38). Associated acceleration and deceleration forces cause shifting and rotation of intracranial contents, resulting in both focal damage and diffuse axonal injury (14; 48). Injury to the brain can occur at the site (coup) as well as opposite to the site of the injury (contra-coup) or where the brain strikes bony protuberances and sharp edges of the internal skull. There is significant heterogeneity in the nature of these injuries (57), such that individuals with TBI subsequently present with a myriad of signs and symptoms of varying severity (25).

It has been well established, and for some time, that people with mild to severe chronic TBI demonstrate significantly reduced accuracy when identifying basic facial expressions of emotion (12; 50; 55). The six basic facial emotions are: sad, angry, disgust, anxious (also referred to as fearful or scared), happy and surprise (35). These can be further categorised with respect to valence – either positive (i.e., happy, surprise) or negative (i.e., sad, angry, disgust, anxious) (12). As deficits in facial affect recognition appear as early as 2 months after injury, this is said to be a direct consequence of the injury itself (40). Evidence for the persistence of recognition deficits in chronic TBI has also been demonstrated up to an average of 15 years after injury (6). A meta-analysis has highlighted that between 13% and 39% of the population with moderate to severe TBI are impaired in facial affect recognition versus 7% of healthy counterparts (4). In addition, this deficit is strongly associated with diminished social integration (5; 26), and decreased competence in social communication according to self-report and reports from close-others (52; 69). Impairments in social cognition in these individuals (36; 39), along with social behavioural and personality changes (39; 60), are said to be more disabling than the enduring physical changes experienced by people living with TBI (25).

### Neural correlates of face and facial expression recognition

Facial affect recognition is subserved by the coordination of an intertwined cortical network, distributed with respect to neural substrate location and timing of activation, and which is yet to be fully understood (1; 17; 58). The expanse of this network makes it highly vulnerable to insult following a TBI. Principal cortical areas involved in what has been termed the facial affect processing network (53) include the occipital cortex (i.e., subserving visual input), temporal and parietal cortices, the limbic system (including the amygdala and hippocampus), and the frontal cortex; namely the orbitofrontal and prefrontal areas (16; 17; 22). The fusiform face area (FFA) in the superior temporal sulcus selectively responds to emotional face content and face identity (21; 22). The FFA is less activated in people with moderate-to-severe TBI who are impaired in interpreting facial emotional expressions (54). Furthermore, reduced performance on facial affect recognition tasks in people with severe TBI has been associated with reduced integrity of white matter tract pathways that connect the occipital and frontal areas, as well as reduced grey matter volume in limbic regions (14).

Facial affect recognition begins with visual input and ends with categorization or labelling of the emotion (1; 2). Three ‘elements’ have been used to describe emotional face processing, with each element linked and progressively building upon the other (43). The first element is the visual perception of the emotional face. Faces are processed holistically and then by separate facial feature analysis (63). The latter is achieved by moving the eyes so that they are oriented to the feature of interest (73). The second element is emotion replication and experience within oneself, including changes in one’s body state and arousal (43). Some researchers have, for example, used mimicry to train emotional processing in persons with TBI in an attempt to improve facial emotion recognition (51). The third and final element involves emotion interpretation which is exemplified through emotional labelling. This element is associated with one’s previous experience of the emotion, including memories and situational contexts within which the emotion was experienced. In this way, a person’s conceptual understanding of the emotion is used to interpret and label it (16). The accuracy of this third element, and thus of recognition performance overall, represents the culmination of the type of information acquired and processed in the previous elements.

### The visual scanpath to faces

A visual scanpath is comprised of a series of foveal fixations and their inter-connecting saccadic eye movements that form during visual search (24). It provides a direct, objective measure of visual attention (49). Eye movements orient the high acuity foveae to fixate a point of interest from where detailed visual information is extracted. Visual scanpaths to faces recorded from healthy controls generally include fixations to the most emotionally-salient facial features – the eyes, nose and mouth (45). However, populations with poor facial affect recognition sometimes show aberrant scanning strategies. People with schizophrenia, for example, demonstrate globally restricted visual scanpaths, comprised of fewer fixations, longer fixation durations, and short saccadic length (30; 71). Contrastingly, people with social phobia show increased saccadic length, or what has been called hyper scanning, with significantly fewer fixations to the eyes on the face stimulus. This has been especially noted for angry faces (19). Autistic individuals have also demonstrated disorganisation in scanning emotional face stimuli, with a preference for looking at non-emotive facial features like the hair-line and ears (47).

The visual scanpath characteristics in TBI are less clear because a dearth of research exists. Mancuso and colleagues (32) found that, while their group of twenty-four adult participants with chronic severe TBI demonstrated significantly reduced face affect recognition, this was not the result of a disrupted scanning strategy. Greene’s doctoral research (15) demonstrated that the visual scanning patterns of people living with mild to severe chronic TBI were disrupted during some tasks of social cognition, even though no statistically significant correlation existed between emotion recognition performance and eye tracking data (15). Taken together, these findings appear at odds with our earlier pilot work (67). We demonstrated that a small group (n = 4) of people with chronic severe TBI and poor affect recognition also demonstrated an aberrant visual scanning pattern. While control participants attended significantly more frequently and for significantly longer periods of time to internal facial features (eyes, nose and mouth), people with TBI demonstrated a widespread visual scanpath pattern. They demonstrated no statistically significant difference in the number and duration of fixations to internal facial features (eyes, nose, mouth) versus external facial features (all other areas) (67). This scanning approach likely affected their ability to extract the most salient visual information from the emotional face stimulus upon which to interpret the emotion shown.

Understanding the visual scanning strategy employed by groups with poor affect recognition is important for many reasons, including that it provides a potential avenue for remediation primarily targeted at the level of visual perception construction. However, studies using eye movement training coupled with real-time recording in such populations are extremely scant. The research has largely focussed on people with schizophrenia, with each study respectively showing no statistically significant association between improvements in facial affect recognition and the underlying visual scanning pattern (11; 34; 56). Some have reported trends at best (56); while others cite the lack of association being the result of poor statistical power (34), and the need for stronger integration of eye tracking into the training paradigm a priori, to more actively direct visual attention (e.g., using visual prompts over salient facial areas) (11). To the best of our knowledge, no other studies have set out to use eye movement training in other cohorts with deficits in facial affect recognition. Although a small number of researchers have piloted various intervention techniques targeted at improving facial affect recognition in people with moderate to severe TBI (7, 8; 42; 51; 70), they do not appear to have recorded or reported upon the nature of the visual scanpath employed by these individuals.

### The present study

We have recently reported on the effect of a combined visual scanning and verbal cuing intervention on emotion recognition accuracy and response times by a person with chronic severe TBI (BR) (66). In that study, BR demonstrated statistically significant improvement in recognition performance to basic facial expressions of emotion following the combined intervention, along with statistically significantly prolonged response times. In contrast, the purpose of the present study was to investigate BR’s visual scanpath to pictures of facial expressions of emotion, including whether his scanpath could be changed for optimal scanning via intervention. Our aims were therefore twofold. Our first aim was to better understand the nature of BR’s visual scanning strategy without intervention. We hypothesized that his baseline scanning strategy would likely be aberrant, but we could not be precise as to the nature of this aberration. Secondly, we sought to determine whether the intervention had an impact on his visual scanpath including whether any changes observed were maintained at a follow up visit. We did not formulate any hypotheses in relation to this second aim.

## Methods

### Participant

BR was a 35-year-old male who had sustained a severe TBI 6 years prior to the study (LOC 7.5 weeks, PTA 90 days). He had completed 19 years of education, reported no familial history of schizophrenia (31), and was strongly right-handed (9). BR demonstrated no evidence of visual neglect when assessed using both the Line Bisection test, and the Albert’s Cancellation task (3). He wore prescription glasses throughout testing which corrected his myopia and astigmatism. Ground-in base in prisms were present in each eye lens, to neutralise the effect of his left exotropia at near. He had normal corrected visual acuity (distant and near; LogMAR chart), colour vision (to Ishihara, and City University Colour Vision Test) and demonstrated full monocular and binocular visual fields (to confrontation assessment, and Humphrey Field Analyser: 30-2 SITA Standard each eye, and Binocular Esterman test (Carl Zeiss Meditec)). BR’s direct and consensual pupillary responses were normal, but he was unable to demonstrate convergence to a near target. His eye movement excursions were full to nine gaze positions when assessed using a pen torch stimulus. His internal ocular health showed no apparent defect on slit lamp and fundal examination.

### Study Design

An AB single case design with follow up was used. It comprised 6 baseline sessions (A), 12 intervention sessions (B) and a single follow up session 3-weeks after the final intervention session. The study spanned 11-weeks in total.

### Stimuli

Seventy-two coloured photographs were selected from the Radboud Faces Database (RaFD) (Langner et al., 2010) with permission for use granted to the first author. Male and female, adult and child Dutch Caucasian faces were selected that displayed one of the following universal emotional expressions (13): happy, surprised, angry, disgusted, anxious, sad. Twelve photographs of each expression were selected where they had a high inter-relater reliability (> 75%) (Langner et al., 2010), and were taken in frontal position only. Two separate stimulus sets were developed, each including 6 photographs of each respective facial expression. One set was used for baseline sessions 2 to 6, with the other set used during each of the 12 intervention sessions. All 72 stimuli were shown at baseline session one and at follow up. Each set was matched for gender, adult, child, emotion, emotional valence (i.e., positive or negative), and average inter-rater reliability which was 94%. Each stimulus was resized to 883 x 1024 pixels (width x height, WxH) to enable its display on the eye tracker monitor.

### Apparatus

BR’s visual scanpath was recorded at a sampling rate of 60 Hz using the Tobii T120 binocular infrared eye tracker (Tobii Pro, Stockholm, Sweden). The fixation number, duration (milliseconds, ms) and location – with respect to pre-defined areas of interest (see below) – were recorded in real time for later off-line analyses using Tobii Studio 3.4.8. The configuration included a double monitor and double keyboard connected to a single computer tower (Dell Precision T3500), with all stimuli presented on an integrated T120 eye tracker monitor (1280 x 1024 pixels). BR was positioned an approximate average distance of 60 cm (range = 58 to 62 cm) from the eye tracker monitor throughout testing, so that the visual angle subtended by the screen was approximately 32º x 24º. No head restraint was used. Two assessors were positioned before the second monitor where they initiated stimulus sequences and actively monitored BR’s fixation. Calibration trials were undertaken at the commencement of each recording session using a 9-point (3 x 3) reference grid and sequence of stimulus presentation (blue moving spot) was automatically generated by Tobii Studio software (62). The software did not permit eye tracking to proceed when insufficient calibration data was acquired at given points. Recalibration of those points was undertaken in these instances by the assessor. In addition, calibration was repeated during sessions when BR made a large movement or needed a rest. The T120 is stated to be accurate to within 0.5º when head movement is minimal and lab conditions remain constant (62).

### Procedure

The study took place in the Eye Movement Laboratory, La Trobe University, Melbourne. Approval was obtained from the Faculty of Health Sciences Human Ethics Committee, La Trobe University. The procedure has been previously described in detail (66). A summary is provided below.

**Figure 1.**
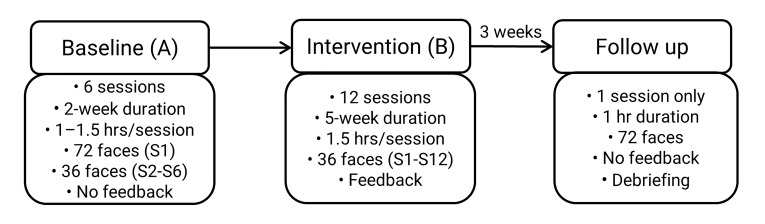
Study Procedure. S = session; hr = hour.

The study procedure from baseline to intervention to follow up is summarised in Figure 1. During each session, apart from when the intervention was applied, BR controlled the pace of stimulus presentation, advancing the sequence by pressing the keyboard spacebar. A central fixation marker was shown prior to every facial stimulus, so that BR viewed each face from the same starting position (65). Each face stimulus was presented for as long as BR required before he decided upon the emotion. He read aloud the emotion from a list provided on the screen which was recorded manually by two independent assessors. BR did not receive feedback about his performance at baseline or follow up. BR received feedback about his recognition accuracy only during intervention.

**Figure 2.**
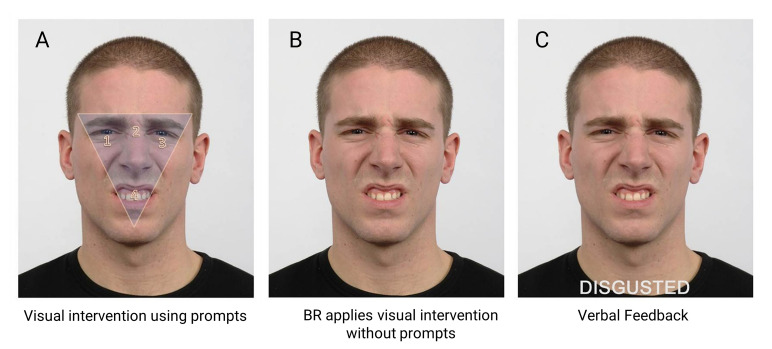
Intervention steps using a disgusted expression. A) Visual intervention: numbered prompts directed BR’s foveal fixations to salient facial features. 1 = left eye, 2 = nasion, 3 = right eye, 4 = mouth. B) Application to same facial expression. C) Verbal intervention with feedback. The emotional label appeared below the face for reinforcement. Note: interstimulus fixation marker and labels not shown.

The intervention combined visual and verbal training. It was only applied when BR incorrectly labelled an emotion during Phase B. The visual scanning component required BR to look at four numbered visual prompts (1, 2, 3, 4. See Figure 2A) located within a shaded inverted triangle that had been superimposed upon the face stimulus he had incorrectly labelled. Reading from a script, one assessor instructed BR as follows, *“*Look at the area inside the triangle when looking at the face. First, look at the eye area, the left eye number 1, at the middle of the eyes, number 2, and the right eye, number 3. Then look at the mouth area, number 4.” BR repeated this scanning pattern three times during which time his ocular position was monitored by two assessors using the live tracker display provided by Tobii Studio (62). BR was then instructed to apply this same approach when looking at the next face – the same face stimulus without visual prompts (Figure 2B) – and press the spacebar when he had decided on the emotion, which he read aloud from a list that was presented immediately following the disappearance of the face stimulus. BR was then shown the same stimulus, with the correct label written in capital letters beneath the face (Figure 2C). At this stage, verbal intervention was provided by one assessor. BR was given structured verbal feedback about his accuracy. For example, if BR’s labelling was correct, verbal reinforcement was applied, “Yes, this person is disgusted.” Correction was however applied for incorrect responses, “That is incorrect. You said this was [insert emotion stated], but it is disgusted.” In both situations, the assessor continued with their feedback and defined the emotion in relation to its: i) valence (i.e., positive or negative), ii) synonym and situational meaning (26), and iii) facial cues that associated the facial expression with the emotion (35). To continue with the current example, this would be, “Disgusted is a negative emotion. It’s how you feel when something is revolting like a really bad smell. In disgust, the eyebrows are lowered, and the eyes may be slightly narrowed. The upper lip is lifted, and this is combined with a wrinkled nose. The upper teeth may be shown because the upper lip is raised.”

Face stimuli were randomised during each study phase. The list of emotional labels was randomised within but not between sessions. Two assessors were present for all except two intervention sessions. Their respective accuracy recordings across each phase yielded 100% agreement. BR was debriefed upon study completion and thanked for his participation.

### Data Analyses

Visual scanpath data were analysed for correctly labelled responses only. This decision was made to control for the inherent differential difficulty in recognition that is reported to occur across universal emotional facial expressions. In healthy cohorts, for example, anxious or fearful facial expressions are one of the least accurately identified emotions, with an accuracy of approximately 30% and most often confused with surprised expressions (e.g., 46). On the other hand, happy facial expressions are likely to yield a less than 1% error (46). In light of this, data were analysed for correctly-labelled responses only so that the level of difficulty of emotion was not a confounding variable.

BR’s visual scanpath parameters were analysed with respect to the mean number of fixations and mean duration of fixations (ms) captured within defined Areas of Interest (AOIs see below). These temporal parameters are customarily analysed in scanning analyses, providing measures of attentional allocation and processing speed. A fixation was defined using the Clearview Fixation Filter (62) set to binocular averaging. A fixation was included when it remained within a 50-pixel area for 100 ms or longer.

### Areas of Interest (AOIs)

**Figure 3.**
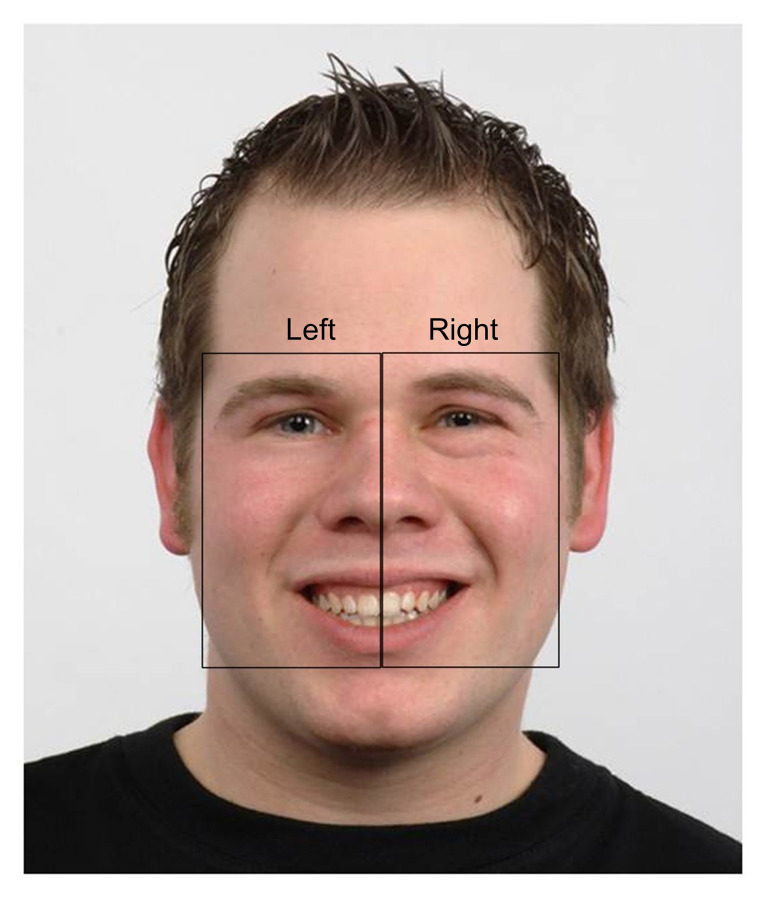
Areas of Interest (AOIs) shown superimposed onto a face stimulus. Note that the right AOI is on the right of the face stimulus and the left AOI is on the left of the face stimulus to align with how BR viewed the stimulus.

The Areas of Interest (AOIs) were defined following qualitative visual inspection of BR’s scanpath across each study phase by both authors. Two rectangular, non-overlapping AOIs – right and left of face stimulus – were then manually constructed by the first author using Tobii Studio 3.4.8 (62) (Figure 3). These ‘bespoke’ AOIs were used because they corresponded with BR’s extremely lateralized scanpath pattern observed at baseline.

Each AOI included (in part) the eyes, nose, mouth and, by virtue of their rectangular shape, the cheeks. Where possible, AOI sizes were equivalent to each other both within and between stimuli. The precise dimensions of the AOIs between stimuli (and sometimes within stimuli) were however largely dependent upon face shape (e.g., adult versus child, face symmetry) and the facial expression (e.g., mouth shape or eyebrow position). Some minor variation thus necessarily existed between expressions with respect to AOI area. Nonetheless, the same facial features were included in each AOI for all stimuli.

Each AOI was constructed slightly larger than the outside edge of facial features, to include an outer margin equivalent to 0.5º of visual angle at BR’s approximate viewing distance (60 cms). This allowed some buffer zone for eye tracker inaccuracy (62). Where offsets in the data were noticeable, this was managed by manually shifting the AOIs an equivalent amount to capture the data. Data offsets are visible when the difference between the actual and measured fixation position is significantly greater than the level of inaccuracy reported by the manufacturer under optimal viewing conditions (62). Data offsets represent an artefact in eye tracking. Offsets occur usually as a consequence of head movement, poor posture (44) or the use of corrective lenses (62).

### Single case design analyses

Data were plotted on a line graph and systematic visual analyses of key variables were undertaken without further quantification, following the method described by Kratochwill et al. (27). The five key variables assessed were: level (mean score for the data within a phase), trend (line of best fit within a phase (33)), variability (range of data points within a phase), overlap (overlapping data points between phases), and the immediacy of effect once the intervention had been introduced (i.e., a phase comparison between the last 3 data points of phase A and first 3 data points of phase B) (27; 28; 33). An AB design with follow up does not permit replication of results in like-phases. Therefore, a functional relation between intervention and BR’s outcomes could not be directly established (28). Both authors analysed each graph which yielded an inter-rater reliability of 93%. Where there was disagreement, discussion ensued until consensus was obtained.

As an adjunct to the visual analyses, the Tau statistic was calculated using a web based application for single case research (64). The mean number and mean duration of fixations within each AOI were analysed between baseline and intervention phases.

The Wilcoxon Signed-rank test was used to compare the mean number and mean duration of fixations to the right versus left AOI within the baseline phase and within the intervention phase. The level of statistical significance was set at *p* < 0.05.

## Results

### Emotions collapsed

BR’s mean number and mean duration (ms) of fixations to each AOI for correct responses were initially analysed with emotions collapsed (Table 1) and then analysed by emotion (Table 2).

### Mean number of fixations

**Figure 4.**
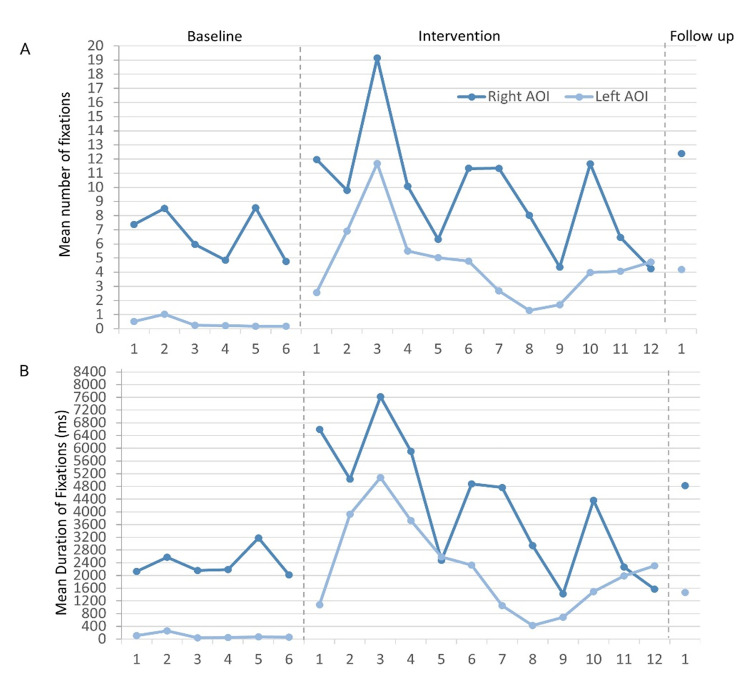
A) Mean number and B) mean duration (ms) of fixations to each AOI across respective phases and sessions.

On visual inspection of graphed data, the mean number of fixations to the right AOI was varied yet stable across baseline sessions (Figure 4A). No statistically significant baseline trend existed (Tau_TrendA_= -0.333, p = 0.355). An immediate effect of the intervention was observed, and BR made the greatest mean number of fixations at intervention session 3 (19.15 + 16.13). There was 100% overlap in the data between baseline and intervention phases, largely because of a combination of variability during intervention (SD = 4.12), and the general decline in mean values after intervention session 3. No statistically significant trend was recorded in phase B (Tau_TrendB_ = -0.394, p = 0.075). BR generated more fixations to the right AOI during the intervention, but this did not reach statistical significance (Tau = 0.444, p = 0.134). BR’s mean number of fixations to the right AOI further increased at follow up (mean = 12.41; Table 1)

**Table 1.** Mean number and duration of fixations (in milliseconds, ms) to AOIs for correct responses. SD shown in parenthesis. *p*-value represents the statistical significance for baseline versus intervention. *p < 0.05.

		Baseline	Intervention	Follow up	*p*-value
Number of fixations					
Right AOI		6.67 (1.73)	9.57 (4.12)	12.41 (9.05)	0.134
Left AOI		0.40 (0.34)	4.58 (2.76)	4.20 (4.61)	0.001*
Duration of fixations (ms)					
Right AOI		2376.83 (437.12)	4155.07 (2016.18)	4827.48 (3721.80)	0.0752
Left AOI		101.42 (82.29)	2224.40 (1423.25)	1471.68 (1666.70)	0.001*

**Table 2.** Statistical values for the mean number and duration of fixations (in milliseconds, ms) to AOIs for correct responses by emotion. *p*-value represents the statistical significance for baseline versus intervention. *p < 0.05.

	Mean number of fixations				Mean duration of fixations (ms)			
	Right AOI		Left AOI		Right AOI		Left AOI	
Emotion	Tau	*p*-value	Tau	*p*-value	Tau	*p*-value	Tau	*p*-value
								
Anger	0.403	0.174	1.00	0.001*	0.333	0.261	0.972	0.001*
Disgust	0.056	0.852	1.00	0.001*	0.028	0.925	1.00	0.001*
Anxious	0.347	0.242	0.917	0.002*	0.361	0.223	1.00	0.001*
Happy	-0.806	0.007*	0.681	0.022*	-0.694	0.019*	0.597	0.044*
Sad	0.556	0.061	0.917	0.002*	0.694	0.019*	0.944	0.002*
Surprise	0.389	0.190	1.00	0.001*	0.472	0.111	1.00	0.001*

BR’s mean number of fixations to the left AOI were only slightly variable (Figure 4A), but a statistically significant negative trend was recorded (Tau_TrendA_ = - 0.800, p = 0.024) – BR made progressively fewer fixations to the left AOI across baseline sessions. BR demonstrated an immediate increase in the mean number of fixations to the left AOI following the introduction of the intervention. Compared with baseline, there was greater variability in BR’s data during intervention. There was no statistically significant trend in this phase (Tau_TrendB_ = - 0.303, p = 0.170), and no overlap in the data between phases – BR did not return to baseline values during intervention. The increase in mean number of fixations to the left AOI after intervention was highly statistically significant (Tau = 1.00, p < 0.001). At follow up, this increase remained, with a mean of 4.20 – an almost 11-fold increase from BR’s baseline mean value (Table 1).

### Mean duration of fixations

BR’s mean duration of fixations to the right AOI were stable at baseline, showed little variability and no statistically significant trend (Tau_TrendA_ = 0.067, p = 0.851) (Figure 4B). An immediate increase in BR’s mean duration of fixations was observed following the introduction of the intervention. BR reached maximum mean fixation duration at intervention session 3 (7621.42 + 6340.42 ms) with subsequent decline thereafter. A statistically significant negative trend in the intervention data was recorded (Tau_TrendB_ = - 0.667, p = 0.003). The data during intervention was highly variable with 100% data overlap with baseline values. After intervention, no statistically significant change in BR’s mean duration of fixations to the right AOI was recorded (Tau = 0.528, p = 0.0752). His mean duration of fixations to the right AOI increased at follow up (Table 1).

BR’s mean duration of fixation to the left AOI was 101.42 ms at baseline (Table 1). Figure 4B shows that his baseline data were stable, there was little variability and no statistically significant trend (Tau_TrendA_ = 0.200, p = 0.573). Immediate change in his mean duration of fixations to the left AOI occurred following the introduction of the intervention. Once again, the peak in the intervention data was reached at session 3 (5075.27 + 5207.63 ms) with values subsequently declining. The variability in the data during intervention was greater than at baseline. There was no statistically significant trend in the intervention data (Tau_TrendB_ = - 0.333, p = 0.131) and no overlap in the data with baseline – BR’s mean duration of fixation to the left AOI remained above baseline values throughout intervention. BR demonstrated a highly statistically significant increase in the mean duration of fixations to the left AOI (Tau = 1.00, p = 0.001) following the introduction of the intervention. This mean increase remained higher than baseline at follow up (Table 1).

Within phases, BR generated a statistically significantly greater mean duration of fixations to the right versus left AOI both during baseline (Z= -2.201, p = 0.028) and intervention (Z = -2.830, p = 0.005).

### Effect of emotional expression

BR’s visual attentional allocation was statistically analysed with respect to emotion. Tau values provided in Table 2 show that, for each emotion, BR generated a highly statistically significant increase in both the mean number and mean duration of fixations to the left AOI after the introduction of the intervention (all *p*’s < 0.05).

For happy expressions, BR generated statistically significantly fewer fixations (Tau = - 0.806, *p* = 0.007) and spent statistically significantly less time looking to the right AOI following intervention (Tau = -0.694, p = 0.019). In addition, although the number of fixations BR generated to the right AOI did not reach statistical significance for sad expressions (Tau = 0.556, *p* = 0.061), he spent statistically significantly more time looking to the right AOI for sad expressions (Tau = 0.694, *p* = 0.019) following intervention.

### Qualitative inspection of the visual scanpath

**Figure 5.**
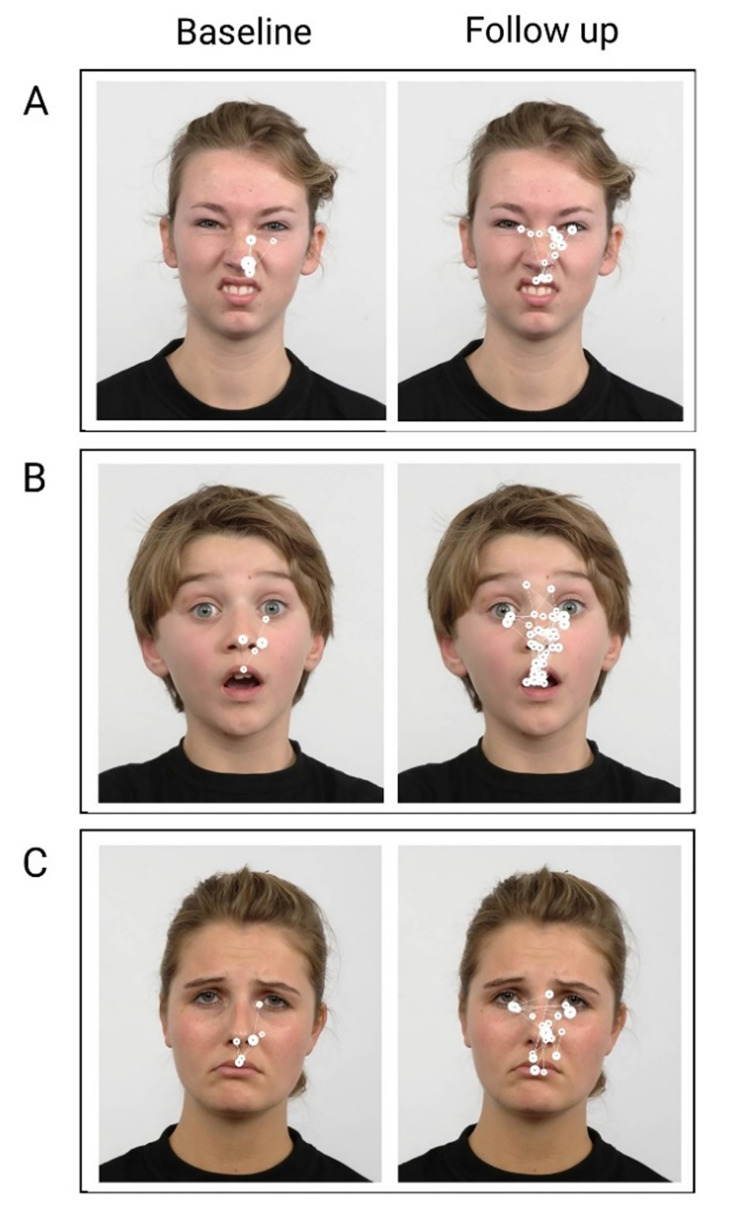
Examples of BR’s visual scanpaths to emotional facial stimuli at baseline and follow up. Filled circles represent foveal fixations and white interconnecting lines represent saccades. Larger circles indicate longer fixation duration. A) Disgust, B) Surprise, C) Sad.

BR’s visual scanpath to selected facial expressions at baseline and follow up are shown in Figure 5. At baseline, BR consistently demonstrated a restricted scanning pattern – in alignment with the quantitative data reported above, his foveal fixations were largely allocated to the right side of the face stimulus. At baseline, he generated fewer fixations directed to salient facial features – i.e., eyes, nose, and mouth – and generally only to the right side of the stimulus. In this way, BR’s scanpath was not only restricted, but also lateralised to his right.

At follow up, BR’s scanning strategy appeared more symmetrical and less restricted. He generated more fixations compared with baseline and to the most salient facial features on both sides of the face stimulus. Overall, BR’s visual scanpath was more lateralised post intervention. It should be noted however that, even at follow up, BR still demonstrated greater attention to the right of the stimulus – more foveal fixations are seen to the right side across emotions – in spite of the increased symmetry in his scanpath at follow up.

## Discussion

This study recorded the visual scanpath that BR, a 35-year-old male with chronic severe TBI, generated to emotional faces before, during and after he underwent a combined visual and verbal feedback intervention. His eye movements were recorded over the course of an 11-week period using an AB design with single follow up. This is the first study to assess the visual scanpath to emotional faces in a person with TBI before and after an intervention paradigm, to use scanpath strategies combined with verbal feedback as an intervention approach and, furthermore, to demonstrate improvement in the visual scanning strategy following intervention. In support of our hypothesis, at baseline BR demonstrated an aberrant visual scanpath and this was characterised by little to no gaze to the left area of the stimulus. BR’s visual attention was largely confined to the right area of interest (AOI). Within the right AOI, however, he did attend to salient facial features, namely the eyes, nose and mouth. BR’s scanpath at baseline resembled half an inverted triangle located within the right AOI. In relation to our second aim, this study has shown that BR’s visual scanning pattern became more lateralised following intervention. BR generated more fixations and spent more time viewing both sides of the stimulus, but this increase was only statistically significant with respect to the left AOI – possibly the result of his greater attentional allocation to the right AOI at baseline. Despite BR’s scanpath becoming more lateralised, his overall attentional bias to the right AOI remained. He generated more fixations and spent more time looking within the right AOI throughout the study, and this was statistically significant both at baseline and during intervention. BR’s preference to look to the right of the stimulus was also recorded at follow up.

Visual perception is the first element in the process of emotional facial recognition (43). This informs the subsequent cognitive elements of emotion replication and experience, and emotion interpretation and labelling or categorisation (43). BR demonstrated disruption at the level of visual percept construction – he acquired what appeared to be incomplete visual information about facial cues upon which to ultimately label the facial emotion. We reported previously on BR’s reduced accuracy in labelling facial expressions of emotion (66), which statistically significantly improved following the introduction of the intervention. We can now extend upon our earlier findings. Taken together, we have demonstrated, and for the first time in a person with TBI, that BR’s poor accuracy in labelling facial expressions of emotion co-existed with his aberrant scanning style and, in addition, that his statistically significant improvement in facial expression recognition following intervention co-existed with the more symmetrical pattern recorded in his scanpath.

Once the stimulus is perceived as a face, saccadic eye movements then orient the foveae to salient features of interest so that visual information of a high resolution can be extracted (68). It has been shown that purposefully restricting eye movements to non-emotional faces in normal cohorts significantly reduces facial recognition accuracy (18). BR’s scanpath pattern at baseline aligns with a basic visual perceptual deficit. In contrast, findings by Mancuso and colleagues (32) suggest that higher-level cortical areas are most likely affected in TBI, because they did not find ‘lower-level’ visual scanning deficits in the presence of reduced affect recognition performance. In our previous pilot work, we recorded hyper scanning in a small group of participants with chronic TBI who also demonstrated reduced facial affect recognition (67). The pathophysiology of TBI is heterogeneous – it involves injury to multiple sites along the facial affect processing network (48), including white matter tracts (14). While our findings contrast with some (15; 32) but not other research (67), the variance between these studies is plausible given the lack of homogeneity in the pathophysiology of TBI.

BR’s visual scanpath differs from visual scanning patterns to emotional faces in clinical groups that have been documented to have facial affect recognition deficits. The visual scanpath has been described as globally restricted in schizophrenia (30; 71), feature-avoidant in social phobia (19) disorganised in autism (47), and focussed on external versus internal facial areas in prosopagnosia (61). While BR was not avoidant of salient facial features per se, his attention was allocated predominantly to the right side of the face stimulus at baseline. BR did not demonstrate a left homonymous hemianopia on visual field assessment, nor did he demonstrate visual-spatial neglect when assessed using the Line Bisection test, and the Albert’s Cancellation Task (3). Inattention is not an uncommon visual disturbance following TBI (23), particularly given that neglect itself is considered a heterogenous syndrome, involving an expanse of right hemisphere areas which is not limited to temporal and parietal areas (41). There is a dearth of studies examining visual scanning patterns in patients with hemispatial neglect, and none appear extant in relation to emotional face stimuli. One report exists of an individual who sustained a focal infarction involving the left inferior parietal lobe (20; 24). Those authors demonstrated that the patient continued to re-scan the right side of stimulus, leading them to postulate that impaired working memory to spatial locations was likely extant in patients with neglect (20). To the best of our knowledge, there is no other report of scanning patterns in TBI that have demonstrated a lateral attentional bias (to faces or other stimuli). Interestingly, Walker-Smith and colleagues (68) have suggested that, because faces are symmetrical, only one side of a face would ever have to be fixated foveally because the whole face could then be internally constructed by the perceiver. It appears, however, that BR benefited from viewing both sides of the face, and without intervention a change to his scanpath would not have been achieved.

No other TBI study to date has used targeted visual scanpath training to improve affect recognition in moderate to severe TBI. The small literature assessing emotional facial remediation in TBI has relied upon remediation approaches using self-instruction training (SIT) (37), and/or errorless learning (EL) (7, 8; 71). Others (42; 51) have guided people with TBI to infer emotions from stories, or trained them to interpret emotionally salient facial cues while drawing upon their somatosensory responses to assist with emotion interpretation. It has been highlighted, at least in the schizophrenia literature, that using visual prompts over salient facial features (i,e., eyes, nose, mouth) during affect recognition training is important in the directing of visual attention, and might result in long-term gains for the individual (11). The use of verbal feedback during remediation paradigms has also been encouraged (51). Both of these approaches were employed in our training paradigm.

There are limitations to this study. We used a pre-experimental single case design with one follow-up visit (10). While BR’s scanpath normalised following intervention, this does not imply that this was a direct result of the intervention – there are many threats to internal validity in a design of this type (10). In addition, BR was not available to participate in more than one follow up visit. It therefore remains unclear whether the impact of the intervention was maintained past the single follow up visit undertaken 3-weeks after the final intervention session, or whether BR’s scanpath parameters returned to baseline values. We recommend that future investigations use a single case experimental design (SCED), with multiple baselines and a longer follow up period to determine maintenance of outcomes (66).

Another limitation to this study, is that it is possible that we missed the presence of visuo-spatial neglect on pre-assessment. We assessed BR using both the Line Bisection and Line Cancellation screening tests (3). The use of a standard attentional test battery has instead been recommended to improve diagnostic sensitivity in cases of neglect (29). Limitations also exist with respect to eye tracker accuracy. The T120 binocular system is accurate to within 0.5º of visual angle (i.e., difference between actual eye position versus recorded position). Greater inaccuracy or data loss can arise from, for example, large head movement, prolonged blinking, squinting or smiling (causing pupillary obstruction), and the use of prescription glasses correction (62). BR wore his prescription glasses throughout the study for optimal visual acuity. While it is possible that this caused inaccuracy greater than the standard 0.5º of visual angle, it is unlikely that the extent of the lateralisation recorded in the scanpath position at baseline was due to BR’s glasses use. BR wore his glasses throughout the study (a constant variable), and a change in his visual scanpath was still recorded across sessions. BR’s head movement could have affected eye tracker accuracy (44). One of the benefits of the Tobii T120 binocular eye tracker is that it does not require participants to be positioned in a chin rest with head restraint (62). However, in the absence of such restriction, sudden or fast head movements (which could arise while the participant is speaking) or poor posture can cause a mismatch between eye position and data position. Data offsets arising from infrared eye tracking are common artefacts to manage (44). The first author is experienced in assessing eye tracking data.

There remains much to be explored in the field of visual scanpath training to emotional faces in people with TBI. Our findings from this work have suggested that face stimuli displaying the happy emotion could be removed from training paradigms. BR demonstrated a statistically significant reduction in the number and duration of fixations to happy faces as the intervention progressed. Negative emotions can be difficult for people with TBI to interpret (12), although this has been discounted by some (55). Furthermore, the contrast in BR’s scanpath with the findings of Mancuso and colleagues (32), suggests that further investigations with larger TBI cohorts are required to map the visual scanpath to emotional faces. It is possible that there are a variety of scanpath patterns that exist in this heterogeneous population and this is yet to be explored.

The findings from this investigation add to our prior work with BR, which focused solely on his accuracy and response times to facial affect interpretation (67). However, a specifically-designed investigation is required to determine the association between accuracy, response time and visual scanpath parameters in people with TBI. In addition, it remains unclear whether scanpaths differ to correct versus incorrectly-labelled expressions of facial effect and indeed whether scanpath differences can be used as a predictor of facial affect recognition. The results from such work are not only of interest in their own right, but could subsequently inform the design of intervention paradigms that have the potential to yield improved performance outcomes.

Consideration could also be given to assessing the effectiveness of how visual scanning interventions translate to real-life situations. Following intervention, assessing the experiences of close-others (69) may provide insight as to whether the person appears to interact and respond differently in social situations. We did not undertake this added analysis with BR, but he did explain to us independently without direct questioning during a non-planned informal interaction that he thought he had improved in determining how others were feeling as a result of his participation in this study. And finally, it is unclear how scanning a two-dimensional emotional face, like the stimuli used in this study, differs from the visual scanning of a three-dimensional emotional face. Further work incorporating more authentic scenarios is also warranted to elucidate scanpath patterns under different conditions.

### Conclusions

This study demonstrated that a combined verbal and visual feedback intervention improved the visual scanpath in BR, a person with chronic severe TBI. Across emotions, BR’s scanpath commenced as one characterised as restricted, with an attentional bias to the right and inattention to the left. Following intervention, it changed to a more lateralised scanpath, resembling an inverted triangle, as per the intervention instructions. However, BR continued to demonstrate a statistically significant attentional bias to the right of the stimulus, as evidenced by a higher proportion and duration of fixations to the right area of interest. Importantly, this study has enabled us to extend upon our previous work (66). Together these results show that, in a person with chronic severe TBI, poor affect recognition performance can coexist with an aberrant visual scanpath and, in addition, that changing the pattern and position of the visual scanpath so that it includes the most salient visual cues can concomitantly improve emotional facial recognition. This study is the first to demonstrate that an aberrant visual scanpath in a person with TBI can be modified through intervention targeted at the level of visual perception construction. These findings contribute to what little is known in the literature about the visual scanpath to facial affect in people with chronic severe TBI. Moreover, this investigation allows the clinician to begin to think about the possibility of including visual scanpath training to their rehabilitation approaches for these individuals.

### Ethics and Conflict of Interest

The author(s) declare(s) that the contents of the article are in agreement with the ethics described in https://bop.unibe.ch/JEMR/about and that there is no conflict of interest regarding the publication of this paper.

### Acknowledgements

The authors thank BR whose enthusiasm and willingness to give so freely of his time enabled this work to be possible. We acknowledge Jessica Boyle who assisted with data collection. We thank the authors of the Radboud Faces Database (RaFD) (Langner et al., 2010) for granting permission to use their stimuli. We gratefully acknowledge the financial support received from the Faculty of Health Sciences, La Trobe University [Research Starter Grant] and from Orthoptics Australia (Victorian Branch).
